# A Combined Rheological and Thermomechanical Analysis Approach for the Assessment of Pharmaceutical Polymer Blends

**DOI:** 10.3390/polym14173527

**Published:** 2022-08-27

**Authors:** Mohammad Isreb, Marianiki Chalkia, Timothy Gough, Robert Thomas Forbes, Peter Timmins

**Affiliations:** 1 School of Pharmacy and Medical Sciences, University of Bradford, Bradford BD7 1DP, UK; 2 School of Engineering and Informatics, University of Bradford, Bradford BD7 1DP, UK; 3 Drug Product Science and Technology, Bristol Myers Squibb, Reeds Lane, Moreton CH46 1QW, UK

**Keywords:** dynamic mechanical analyzer, DMA, shear rheometer, pharmaceutical polymers, hydroxypropyl cellulose, hydroxypropyl methylcellulose, mechanical properties, rheology

## Abstract

The viscoelastic nature of polymeric formulations utilised in drug products imparts unique thermomechanical attributes during manufacturing and over the shelf life of the product. Nevertheless, it adds to the challenge of understanding the precise mechanistic behaviour of the product at the microscopic and macroscopic level during each step of the process. Current thermomechanical and rheological characterisation techniques are limited to assessing polymer performance to a single phase and are especially hindered when the polymers are undergoing thermomechanical transitions. Since pharmaceutical processing can occur at these transition conditions, this study successfully proposes a thermomechanical characterisation approach combining both mechanical and rheological data to construct a comprehensive profiling of polymeric materials spanning both glassy and rubbery phases. This approach has been used in this study to assess the mechanical and rheological behaviour of heterogenous polymer blends of hydroxypropyl cellulose (HPC) and hydroxypropyl methylcellulose (HPMC) over a shearing rate range of 0.1–100 s^−1^ and a temperature range of 30–200 °C. The results indicate that HPC and HPMC do not appear to interact when mixing and that their mixture exhibits the mechanistic properties of the two individual polymers in accordance with their ratio in the mixture. The ability to characterise the behaviour of the polymers and their mixtures before, throughout, and after the glassy to rubbery phase transition by application of the combined techniques provides a unique insight towards a quality-by-design approach to this and other polymer-based solid dosage forms, designed with the potential to accelerate their formulation process through obviating the need for multiple formulation trials.

## 1. Introduction

Polymers find many uses in pharmaceutical products, including acting as bulking agents and binders to enable the granulation of powders, as disintegrants, aesthetic (non-functional) or functional coatings for tablets, and modifiers of drug release from dosage forms. They can also be employed as film forming agents for mouth dissolving medicated strips and as stabilisers of amorphous forms of normally crystalline drugs, to improve solubility and subsequently bioavailability [1,2,3]. The reason for the popularity of polymers is that these unique materials offer a variety of favourable properties such as enhancing stability, providing protection from moisture and light, enabling processability, and also being employed to modify pharmacokinetics and biological activity [4]. However, the recent growing demand to achieve quality by design means that the properties and performance of excipients must be adequately understood to help an informed approach to process development and to predict the short and long-term performance and stability of dosage forms.

Contrary to simple organic small molecule compounds (such as pharmaceutical drug substances), polymers are neither ideal elastic solids nor viscous liquids but are viscoelastic materials. This means that their behaviour is time, temperature, and shear rate dependant. In other words, at any temperature, polymers may behave as solids or liquids depending on the time scale and/or the rate of chain deformation (strain rate) [5]. Several pharmaceutical processes that involve polymers, such as film coating, spray drying or hot melt extrusion, involve processing at elevated temperatures, therefore, it is essential to understand the behaviour of polymers at the temperature and shear rate used in such pharmaceutical processes. Moreover, it is vital to understand the chain relaxation dynamics of the polymer over the shelf life of the dosage form as these could potentially change the physical dimension of the product (e.g., die swelling, creep [6], and film defects such as bridging), its mechanical properties (e.g., tensile strength), as well as its stability and/or the release profile (e.g., film cracking) [7].

Until recently, pharmaceutical processing parameters were usually selected using a trial-and-error approach without necessarily employing a comprehensive understanding of the viscoelastic behaviour of the polymer over the precise conditions of the various stages of the process. For a typical pharmaceutical process, polymers are required to exhibit various distinctive viscoelastic behaviours at each step. For example, in hot melt extrusion, polymers are required to soften, coalesce, and flow within the time, temperature, and shear force exerted in the extruder. However, once outside the extruder the extrudates are expected to be tough and strong (i.e., predominantly elastic) to withstand further processing and handling stresses. Nevertheless, most typical pharmaceutical processes are carried out at relatively low temperatures (below 200 °C) to avoid the degradation of the active pharmaceutical ingredients (APIs). Meanwhile, most practical pharmaceutical polymers are amorphous thermoplastic materials. Therefore, they exhibit no sharp thermal transition from solid to liquid phases. Instead, the polymers transform gradually from predominantly elastic materials to materials dominated by their viscous moduli over a range of temperatures (the midpoint of which is referred to as the glass transition temperature, T_g_) which vary significantly with shear rate and humidity [8]. 

To impart the desired handling properties that help process pharmaceutical polymer formulations in a relatively narrow temperature range, polymer blends and additives are frequently used in the pharmaceutical industry. The impact of these additives is usually characterised using a variety of thermal and mechanical techniques aimed at understanding polymer behaviour at very specific distinctive conditions. These techniques include intrinsic viscosity measurements [9], mechanical strength testing [10,11,12], incidence of stress-induced defects [13], glass transition measurements [9,14], softening temperature [15], and rheological properties [16]. 

The aforementioned techniques are useful for the processes that take place in one phase (i.e., either above or below the T_g_ of the formulation) or at one constant shear regime. For example, the mechanical properties of polymers in their glassy and glass/rubber transition states are usually measured using solid mechanics instruments, such as uniaxial tensile machines or dynamic mechanical analysers (DMA) [17]. Parameters such as tensile strength, elastic modulus, and tan delta (defined as the loss modulus/storage modulus of the sample) are the main parameters usually measured in that case. On the other hand, shear and extensional rheometers are usually used to characterise polymeric formulations in their rubber/liquid and liquid phase [16]. Storage (elastic), loss moduli, tan delta, and complex viscosity are the main measurements to be obtained and analysed. However, the transitional phases between rubber to rubber/liquid are on the borderline of the accuracy limits of the two techniques and are harder to measure accurately and thus be modelled. 

Many pharmaceutical processes occur at conditions at or near the transitional phases of the polymers whilst operating at temperatures that avoid thermal degradation of the drugs. Therefore, it is hard to obtain a comprehensive picture of the polymer performance using a single technique from those mentioned above.

To our best knowledge, there has been no previous attempt to bridge the results from both solid mechanics and rheology into one continuous model. In fact, insufficient work has been carried out to model and understand the behaviour of pharmaceutical polymers and their blends in either the glass or the rubber phases. Therefore, it is vital to build up such knowledge if quality-by-design formulations and processes are to be adopted for optimising pharmaceutical processes.

This study aimed at investigating the feasibility of harmonising the mechanical and rheological data, obtained from DMA and a shear rheometer over a wide range of temperature and shear forces typically encountered during the processing and shelf life of pharmaceutical products. This harmonisation will help produce a continuous and comprehensive master profile to predict the behaviour of two cellulose-based polymers commonly used in the pharmaceutical industry, namely hydroxypropyl cellulose (HPC) and hydroxypropyl methylcellulose (HPMC). Moreover, the effect of polymer blending on the chain dynamics of these polymers will be investigated over the whole range of temperature and shear forces. This will help in the understanding of the extent of interaction between the two polymers and the overall effect of their blend on future solid dosage form design. HPMC polymers are frequently used as drug carrier matrix [18]. However, they are known to have considerably high viscosity in the temperature range between the T_g_ and degradation temperature especially those with higher molecular weight (above 10,000 Da) [19,20]. Therefore, HPMC is often mixed with other polymers or plasticisers to ease processibility [18,19,21]. HPC/HPMC mixtures have been used in hot melt extrusion [22,23] and matrix tablets [24] to exploit the thermoplasticity of HPC [16] and the added functionality of HPMC (e.g., to modify drug release, increase tensile strength, etc.) [21]. However, a proper understanding of the effect of blending these polymers has rarely been investigated beyond the T_g_ value. Indeed, changes in T_g_ values and comparing the calculated solubility parameters could be used to determine the miscibility of two polymers. Mixing two polymers could lead to the formation of a product with multi heterogenous domains, rich in one of the two polymers, and subsequently impacting the manufacturing process (e.g., heterogenous flow, heat transfer, drug solubility) and final product attributes (e.g., mechanical properties and drug loading, distribution and release profile) [25].

## 2. Materials and Methods

### 2.1. Materials

Hydroxypropyl cellulose (HPC), average molecular weight 850,000, viscosity of 2% aqueous solution 4–6.5 mPa (Klucel™ MF, Ashland, Inc., Covington, KY, USA), and hypromellose 2208 (hydroxypropyl methylcellulose, HPMC), average molecular weight 1,000,000, viscosity of 2% aqueous solution 100 Pa.s (Methocel™ K100M, Colorcon, Orpington, UK), were acquired from Dow, UK. Ethanol (reagent grade) was purchased from Fisher Chemical, UK and ultra-purified water (18.2 MΩ·cm) was produced using a Triple Red Laboratory Technology filtering system (Avidity Science, Aylesbury, UK). 

### 2.2. Preparation of Free Films

Free films of the polymers and their blends were prepared to enable characterisation. The blends were prepared by weighing each polymer and mixing them by manual tumbling for 5 min using a zip-locked bag. The powder occupied less than a quarter of the volume of the bag and the rest was filled with trapped air to facilitate mixing. Two methods were used in the preparation of the films for characterisation.

### 2.3. Compressed Discs Preparation

Compressed discs of HPC and HPMC and their blends (Table 1) were prepared using a thermal hydraulic press preheated to 210 °C. A sample of 25 g of the polymer powder (or a pre-blended mixture of the two) was oven dried at 60 °C for 2 h to help reduce risk of film cracking due to moisture evaporation during compression. The dried powder was placed on the surface of the plates in a thickness of approximately 2 mm and then compressed by applying a 20,000 kg force for 10 min using a 0.5 mm spacer. The hydraulic pressure was then released gradually over one minute to avoid cracking due to sudden elastic die swelling. The compressed discs were cut to rectangular shaped samples for DMA measurement and the dimensions were as follows: length: 23.0 ± 1.00 mm (the effective length is controlled by the clamp size of the DMA), width: 3.0 ± 0.05 mm, thickness: 0.5 ± 0.05 mm. For shear rheometer, a 30 mm square was cut (effective shape after trimming is the 25 mm circle of the upper plate).

### 2.4. Casting Polymer from Solution

In order to assess the effect of film compactibility on the rheological measurements, cast films of both pure HPC and HPMC films were prepared. Blends of polymers were difficult to cast as it was challenging to dissolve both HPC and HPMC in a common solvent with a reasonable concentration of both polymers for casting. Hence for cast films, only pure single polymers were evaluated. HPC solution was prepared using an ethanol: water mixture (85:15 *v*/*v*) at a solid ratio of 2% *w*/*v*. Polymer powder was added gradually to the solvent mixture under constant stirring to avoid agglomeration. Films were then cast onto an acetate sheet placed over levelled glass petri dishes and left to dry in a fume cupboard. Successive additions of polymer solutions were needed to build up the thickness of the dry film to 0.5 mm (±0.5 mm). The drying of the film was controlled by covering the petri dish with aluminium foil with pin-holed pores to reduce the evaporation rate over the first 24 h to avoid wrinkling of the film surface and build-up of internal stress [26].

HPMC solution was prepared in water at a solid ratio of 1.5% *w*/*v*, using water at 80 °C when introducing the polymer. Unlike the behaviour in water at room temperature, in hot water, HPMC does not readily hydrate and is practically insoluble, therefore it is less likely to form agglomerates (dry powder with a partly hydrated polymer skin around them) making dissolution difficult. Once the polymer was dispersed in hot water, the temperature was reduced gradually under constant stirring until all the polymer was dissolved [27]. The petri dish was stored in vacuum oven at 30 °C and at a partial vacuum pressure of 0.3 bar overnight. The films were then left to equilibrate at room conditions (20 °C and 65% RH) along with the rest of the films. The films were cut into rectangular strip samples for DMA measurements with the following dimensions: length: 23.0 ± 1.00 mm (the effective length is controlled by the clamp size of the DMA), width: 3.0 ± 0.05 mm, thickness: 0.5 ± 0.05 mm. For the shear rheometer, a 30 mm square was cut and used (effective shape after trimming is the 25 mm circle of the upper plate).

### 2.5. Thermal Gravimetric Analysis

Thermal gravimetric analysis (TGA) testing was carried out using a Q500, Discovery series instrument (TA Instruments, New Castle, DE, USA) to measure the moisture content and the decomposition temperature of the starting materials. Compressed polymer samples (weighing 15 ± 1 mg) were cut for analysis using a cork borer size 2 (5.8 mm in diameter) and evaluated at a heating rate of 10 °C/min. using nitrogen gas atmosphere. The temperature range used was 30–400 °C and the gas flow was set to 25 mL/min. All measurements were carried out in duplicate and analysed using TRIOS software (TA Instruments, New Castle, DE, USA). The determination of the decomposition onset value is essential to identify the temperature range to be used in the differential scanning calorimetry (DSC) test. This helps in avoiding sample decomposition during DSC tests. The degradation temperature was measured just before the onset of the transition peak.

### 2.6. Differential Scanning Calorimetry

DSC was performed using a Q2000 calorimeter (TA Instruments, New Castle, DE, USA) with a refrigerated cooling system. Compressed polymer samples (weighting 15 ± 1 mg) were cut for analysis using a cork borer size 2 (5.8 mm diameter) and enclosed in an aluminium pan with a pin-holed lid to allow moisture evaporation. A heat/cool/heat cycle was carried out for all the samples at a ramping rate of 10 °C/min heating and 5 °C/min for the cooling cycle.

### 2.7. Dynamic Mechanical Analysis

DMA (Q 800, TA Instruments, Newcastle, DE, USA) was used in this study to perform two separate sets of experiments. Firstly, DMA was used to determine the T_g_ of the compressed films. Secondly, the mechanical properties and the dynamic moduli of the polymer or polymer blend preparations were measured. In both tests film strips cut from both compressed and cast films (23 mm × 3 mm × 0.5 mm) were evaluated using an 8 mm long dual cantilever clamp. 

The details of the two methodologies used to measure the samples are presented below.

#### 2.7.1. DMA Methodology for T_g_ Measurements of the Films

Thermal transitions were measured using a temperature sweep test at a fixed frequency of 1 Hz, 0.1% strain, and a temperature ramp rate of 3 °C/min. Loss and storage moduli were measured. The results were analysed using “Universal Analysis 2000 version 4.3A” software from TA instruments. Moreover, the ratio of viscous modulus to elastic modulus at a given frequency (known as tan delta) was calculated using the following equation:

***tan***(***δ***) = ***G″***/***G′*** (1)

Tan delta is widely used to quantify the balance between energy loss and storage in the material. When ***tan***(***δ***) is greater than unity, the material has more viscous properties or plasticity, whereas a ***tan***(***δ***) value lower than unity means more elastic properties, regardless of the viscosity [5].

#### 2.7.2. DMA Methodology for Dynamic Moduli Measurements

To mimic the measurements of complex viscosity in a shear rheometer, DMA was used in a frequency sweep mode to measure the storage and loss moduli using a dual cantilever clamp. Frequency sweep tests were carried out for both compressed and cast films. Initially, a strain sweep test was carried out to determine the linear viscoelastic region of the strain range. Afterwards, frequency sweep tests were carried out using a constant strain value of 0.1%. The temperature was adjusted to 30 °C, held isothermally for 3 min, and a frequency sweep was carried out over a frequency range of 0.1–100 Hz. The temperature was then raised by 10 °C and the cycle was repeated until the sample deformed and the test was disrupted. The loss modulus, storage modulus, and damping factor were recorded and complex viscosity was calculated according to Equation (2) [6]:

(2) $$\eta^{*}=\frac{G^{\prime \prime }}{\omega }+i\frac{G^{\prime }}{\omega }$$

where:

η* = complex viscosity

***G*′***=* storage or elastic modulus

***G*″***=* loss modulus

***ω*** = angular frequency

***i*** = square root of −1

### 2.8. Shear Rheometer Procedure

An oscillatory strain amplitude test was carried out using a Physica MCR 501” rheometer (Anton Paar, Graz, Austria). Parallel plate (diameter = 25 mm) configuration was used to perform all the tests. The gap between the two plates was adjusted to 0.55 ± 0.5 mm (depending on the film thickness). Strain amplitude tests (not shown here) were carried out to determine the linear viscoelastic region (LVR) in order to select the strain value to be used subsequently. Frequency sweep tests were performed using 3% strain and a frequency range of 0.01–100 rad/s. Compressed and cast films were measured at a temperature range of 140–230 °C. Storage, loss and complex viscosities were recorded.

### 2.9. Combining DMA and PPSR Data

Viscosity, referring to the resistance of the material to flow, is usually measured by rheometers and is used to describe the viscoelasticity of polymers in their melt state (above their T_g_). On the other hand, DMA is usually used to evaluate the thermomechanical properties of the material at a given temperature below and around its T_g._ Compared to other thermal analysis methods, such as DSC, DMA provides information about major transitions as well as the more subtle events caused by a side group rotation and/or stretching [28,29]. Since most pharmaceutical processes involve both shear and temperature changes, the thermomechanical and rheological behaviour of the polymers is more relevant than the calorimetric transitions measured by DSC in studying the behaviour of the polymers [5,6,28,30,31,32]. Moreover, DMA is reported to have approximately double the sensitivity of DSC when measuring thermal transitions of polymers [33]. 

Since pharmaceutical processes occur mostly at the transitional boundaries between the two phases in which the polymer transforms between its glassy and rubbery states, this study investigated the possibility of using complex viscosity to express the viscoelasticity of the polymer over both the elastic and the rubber phases. To do this, some assumptions had to be made to simplify the process and allow for data harmonisation. First, this study assumed that thermoplastic polymers in the glassy state are very viscous liquids that will flow over an infinite time scale. Moreover, although the bending movement of the DMA is not a pure shear process, it was assumed to be directly proportional to the shearing movement in the PPSR. These assumptions allow for a direct comparison between complex viscosity values calculated from the dynamic moduli measured in both the PPSR and DMA techniques and therefore a plot of continuous viscosity versus temperature can be constructed across a temperature range spanning from room temperature to well above the T_g_ of both polymers. The frequency of oscillation in both tests was expressed in terms of linear frequency according to Equation (3):

(3) ω=2πf

where ω is the angular frequency in rad/s and ***f*** is the regular frequency and is given by s^−1^.

In order to reduce the data in the two simplified 2D graphs, power law model parameters were used to compare the complex viscosity data of polymers [16,34]. The advantage of using power law is that it reduces the information in the complex viscosity graph over a range of frequency to two numbers: ***K*** and n. Therefore, it was decided to adopt these parameters to allow for the comparison between the data measured by the DMA and PPSR techniques over the whole temperature range. The complex viscosity data from both the DMA and PPSR therefore were fitted to a power law trend line. The power law formulation can be represented by Equation (4):

(4) $$\eta =K{\dot{\gamma }}^{n-1}$$

where η is the complex viscosity of the sample, $\dot{\gamma }$ is the shear rate, ***K*** is the consistency coefficient which describes the complex viscosity value (calculated from the fitted line) at a frequency value of 1 s^−1^, ***n*** is the power law index which decreases as the shear sensitivity of the polymer increases. Therefore, ***K*** and n could describe the complex viscosity of the material over the measured frequency range at a specific temperature. 

## 3. Results and Discussion

Thermal and rheological measurements were used to analyse the effect of blending HPC and HPMC as well as to establish the feasibility of reconciling DMA and PPSR data.

### 3.1. Thermal Gravimetric Analysis (TGA) of the Polymers

**Figure 1:**
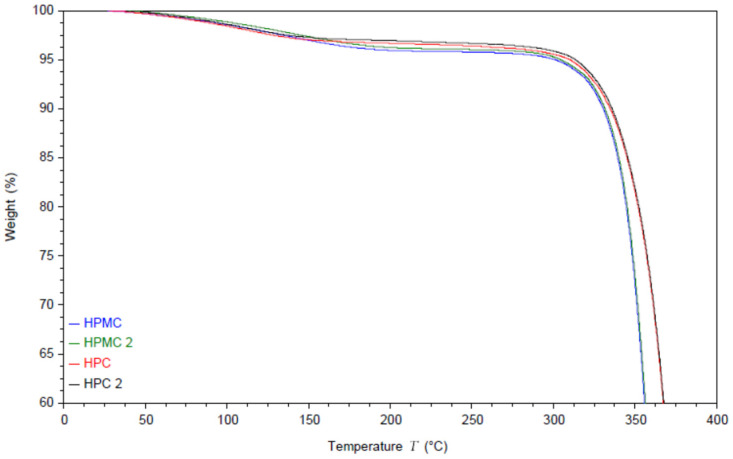
TGA thermogram of two HPC and two HPMC films and the powders. Samples named HPMC and HPC are measured from the powder while HPMC2 and HPC2 are measured from the compressed films.

The moisture content and the thermal degradation of the compressed films were studied using thermal gravimetric analysis (TGA) under nitrogen atmosphere. The results are shown in Figure 1. The thermograms of both polymers exhibit an initial drop in weight due to evaporation of the residual moisture. 

Interestingly the moisture evaporation appears to extend to around 140 °C for HPC films and to around 200 °C for HPMC. The moisture content appears to be around 3% for HPC films and around 4% for HPMC films [35]. It is worth noting that this is the equilibrium moisture content of the films rather than the powder, as the powder was dried in an oven at 80 °C for 2 h before pressing the films [23]. Compressing the powder without the drying step caused cracking in the films. The degradation step of HPMC under nitrogen atmosphere starts at 300 °C [36] while HPC degradation starts at 325 °C [37].

### 3.2. Thermal Analysis of the Films Using DSC and DMA

**Figure 2:**
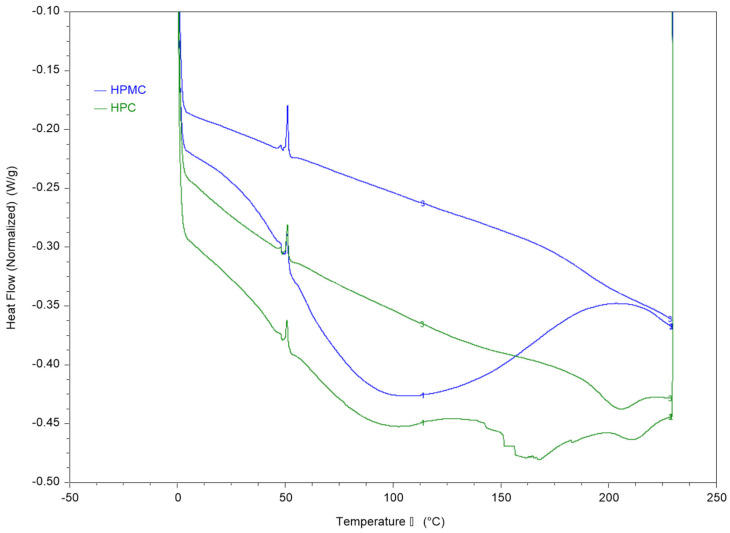
DSC thermograms of HPC and HPMC compressed samples using heat-cool-heat cycle. The figure shows the two heating cycles. The cooling cycle is not shown.

**Figure 3:**
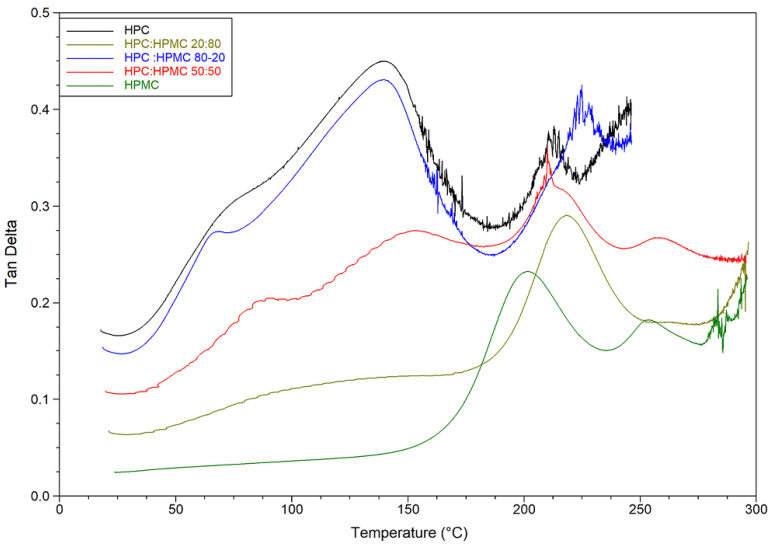
Temperature sweep of tan delta graphs of HPC, HPMC, and their mixtures.

Thermal analysis of HPMC and HPC films and their blends was conducted using DSC and DMA and the results are presented in Figure 2 and Figure 3.

The first heating cycle of both polymers reveals a wide thermal transition between 50–150 °C which is consistent with the moisture loss in the TGA thermogram. The second run allows measurement of the films after moisture evaporation in the first run through the pin-holed pans. The results reveal a T_g_ value of around 187 °C for HPMC and 193 °C for HPC films.

DMA temperature sweep tan delta graphs of the films are presented in Figure 3 and Table 2. The figure reveals that for HPC the ratio of the viscous to elastic moduli (tan delta) peaks at around 140 °C just before the loss of moisture. The ratio drops afterwards (indicating an increase in overall elastic behaviour of the sample) before peaking again at 215 °C. On the other hand, it seems that the moisture content of the HPMC film is not having a noticeable plasticising effect. The tan delta signal for HPMC only peaks at around 216 °C. The mixtures are behaving according to the ratio of HPC to HPMC. The mixture with 80% HPC shows the same peak around 140 °C as pure HPC films. The value of this peak however decreases in the other mixtures as the ratio of HPC decreases. It seems that the mixtures are retaining the characteristic peaks of both polymers and the overall behaviour depends on the ratio of both polymers. Finally, it is worth noting that the hysteresis in the HPC-rich thermograms is due to the softening and sagging of the samples above the glass transition temperature of HPC. Similar behaviour of HPMC-rich thermograms can be seen above 275 °C.

DMA has been reported to record a higher mechanical glass transition temperature than the calorimetric value measured by DSC [38]. This can be attributed to the kinetic nature of the glass transition event which relies on multiple factors including the measurement technique, heating rate, as well as sample history. The T_g_ was measured in the second cycle of heating in the DSC (i.e., after the evaporation of the moisture and with loss of its plasticising effect during the first cycle) whereas the DMA measured the T_g_ of the sample while heating it for the first time (with not enough time for the moisture to evaporate fully). Moreover, the samples are larger in the DMA (a smaller surface area and therefore water evaporation is slower). It is simply not feasible to measure the T_g_ of the polymers in the DSC using the first heating cycle as the moisture evaporation endothermic peak is significantly more prominent and obscures the subtle T_g_ step. This gives an advantage to the DMA in its ability to characterise the mechanical behaviour of the sample without altering its thermal history and therefore represents the actual behaviour of the sample that is expected to be encountered during the formulation process.

It is worth noting that, although the DMA data shows mixing between the two polymers in the blends was such that the domains of each individual polymer existed, it reveals little information about the overall behaviour of the film below or above the single or double transitions noticed in the films. The powder of the two polymers was thoroughly mixed prior to compressing the films, but an ideal mixture was unlikely to have been achieved. Therefore, the presence of individual polymer phases could be also partially explained by lack of ideal mixing. The samples, however, are a more realistic representation of the mixing that can be expected in pharmaceutical processing such as granulation or extrusion and the results therefore are representative of the interaction that would take place during such processes.

### 3.3. Rheological Results

An amplitude sweep test was carried out to determine the strain range through which a linear response is maintained (known as LVE range). In this region the sample deformation is reversible, and the samples behaved in a repeatable manner. From the results, it was decided to use a strain value of 3% for all oscillatory tests in the rheometer and 0.1% strain was used in the DMA experiments.

**Figure 4:**
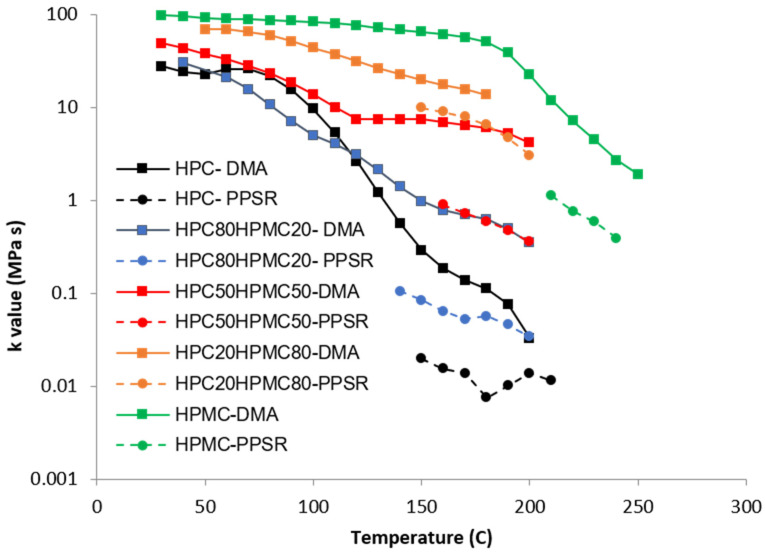
Temperature dependency of the ***K*** value of the power law model of the complex viscosity of the HPC and HPMC mixtures measured by DMA and PPSR.

**Figure 5:**
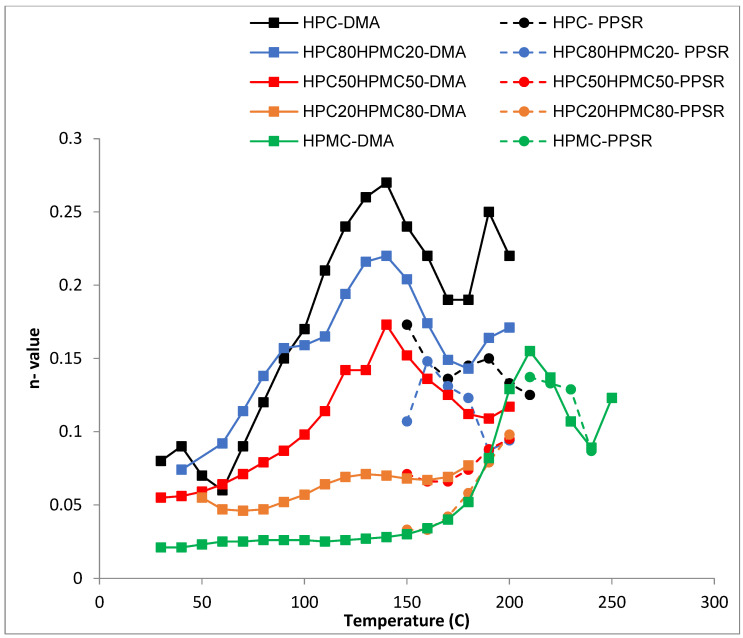
Temperature dependency of ***n*** value (shear sensitivity index) of the power law model for HPC and HPMC mixtures measured by DMA and PPSR.

Complex viscosity data from both the DMA and PPSR were measured and power law model was used to model the complex viscosity graphs from each instrument at each temperature step. Power law parameters—the consistency coefficient ***K*** and the shear sensitivity index (***n***)—were calculated from the equation and plotted against the temperature for each film in Figure 4 and Figure 5. Each single ***K*** value (Figure 4) represents the value of the average complex viscosity at 1 Hz for each graph at each temperature step. As expected, the figure reveals that, as the HPC ratio in the film drops, the complex viscosity increases. This is expected as HPC has a more pronounced thermoplastic behaviour than HPMC [23]. However, it is interesting to note that the viscosity of HPC compressed films starts to drop significantly faster than all other films above 80 °C. The rate of reduction in the viscosity value slows down closer to 140 °C which is the temperature at which the first transition was noticed in the DMA temperature sweep. Importantly, each film exhibits a unique behaviour that cannot be inferred from the Tg value presented in the DSC or the DMA. This illustrates the importance of measuring the mechanical and rheological behaviour of the film at the required shear range at each temperature.

Since no single instrument can perform this measurement over the whole temperature range, it is essential to combine the data from more than one technique as attempted in this study.

The shear sensitivity (Figure 5) of all the films decreases sharply (higher n value) with increasing temperature up until 140 °C. The exception is the film with ≤20% HPC content. This is in line with the Tg data from the DMA. Moreover, all the films reveal another reduction in their shear sensitivity around 180 °C as the HPMC component softens when the temperature approaches its Tg value (Figure 5). This finding again supports the suggestion that the two polymers are not completely mixed with each other as the characteristic of each polymer is visible in the mixture according to the ratio of each polymer.

Most importantly the complex viscosity data from the DMA and PPSR seems to be following the same trend with a correlation factor of 12 for ***K*** values of HPC-rich films and 1.5 for ***n*** values of all films (Table 3).

The difference in the ***K*** values between the DMA and PPSR could be due to the fact that both instruments are at the limit of their ability to reliably make measurements for the samples. In DMA samples start to expand, soften, and sag between the clamped areas as the temperature increases which means the measurements are no longer within the LVE region. Likewise, the PPSR starts to struggle to control the amplitude of the rotational shearing as the sample hardens when the temperature is decreased. Moreover, the difference might also be because the polymer particles had not fully coalesced when they were compressed to make the film. If this assumption is correct, then the DMA measurements would not affect the degree of coalescence because it operates below the Tg value of the polymers. Meanwhile the PPSR is expected to promote particle coalescence and chain entanglement through the application of shearing force and because the film is in the rubbery state. 

**Figure 6:**
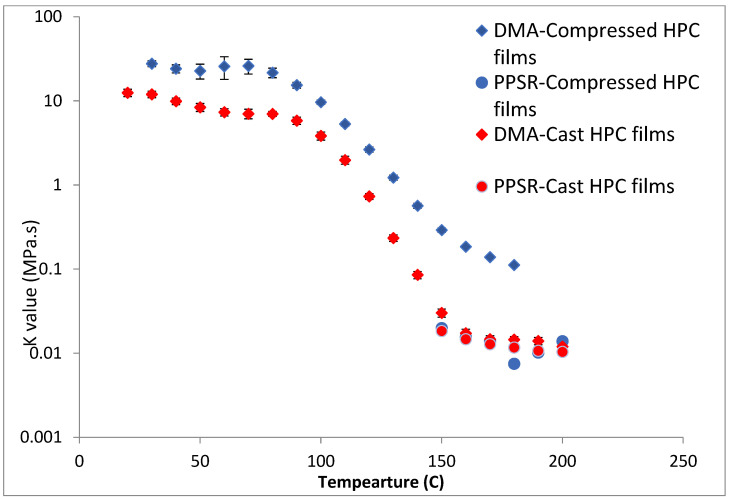
Comparison of ***K*** value of power law model for compressed and cast HPC films using complex viscosity data measured by DMA and shear rheometry. Each point represents the average ***K*** value of three samples and error bars are represented as ± standard deviation.

**Figure 7:**
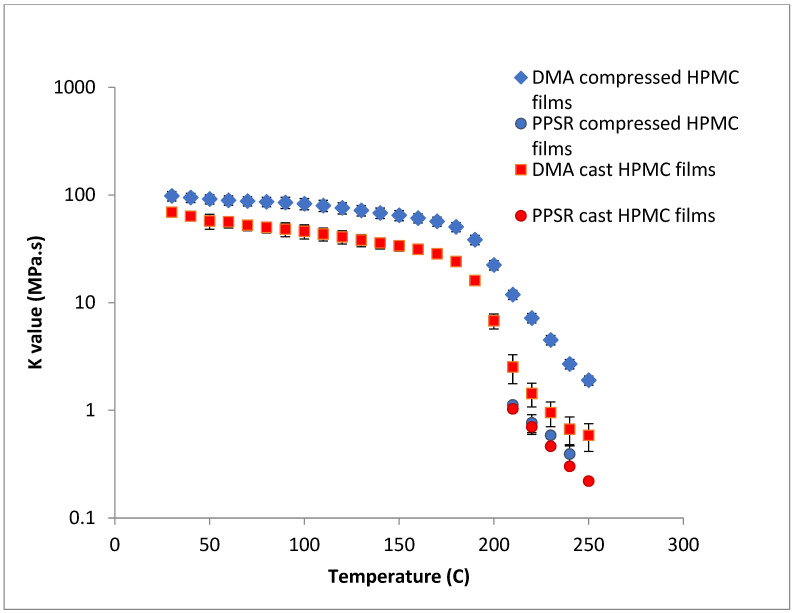
Comparison of ***K*** value of power law model for compressed and cast HPMC films using complex viscosity data measured by DMA and shear rheometry. Each point represents the average ***K*** value of three samples and error bars are represented as ± standard deviation.

To test this hypothesis cast films of HPC and HPMC were prepared and and compared to the compressed films as demonstrated in Figure 6 and Figure 7. It is clear that PPSR data has not been affected by the preparation methodology while as predicted DMA data has changed. Indeed, the viscosity values measured using the compressed films were higher than those measured from the cast film. This might be due to the friction forces between the incompletely coalesced particles of the powder. The DMA dual cantilever exerts a complex stress profile combining compression, tensile, and shearing elements which is different to the shearing forces in the PPSR. In fact, the cast film results for DMA and PPSR are practically equal in values of ***K***. In order to verify that these results were not due to sample error, the measurements were repeated three times and the average ***K*** value was plotted with the standard deviation as the error bar (average ± SD). 

In the case of HPMC, the difference between the DMA and PPSR measurements for the cast film was reduced to 3 (compared to a difference ratio of 9 in the case of compressed films). The remaining discrepancy might be due to the thermal expansion of the sample in the DMA as it is heated from 30–250 °C compared to 30–200 °C for HPC. This could explain the relatively higher standard deviations of the HPMC measurements above 200 °C in the DMA. Moreover, the highly elastic nature of HPMC compared to HPC made it harder to measure the sample in the PPSR technique. In fact, the PPSR raw data file usually shows a “DSO” status when the test is carried out reliably indicating that the measurement was taken by applying the exact strain in the oscillatory test. This was the case for the HPC samples. In the HPMC samples however the file consistently displayed the status “WMa” which suggests that the machine stopped attempting to apply precise strain after multiple failed attempts. In addition the normal force was consistently higher for HPMC samples compared to HPC due to the Weissenberg effect of the more viscous HPMC samples [39]. The latter is another sign that HPMC samples were not relaxing back after each measurement. It is worth noting that the error bars (measured as the ±SD of the three measurements) were still relatively small for HPMC samples suggesting consistent acceptable error in the various measurements. In essence, the film sample of HPC softened when measured with PPSR allowing the surface of the film to adhere to the plates and thus eliminating gaps between the film and the plates and providing appropriate grip for the shearing force of the upper plate to exert the precise deformation. On the other hand, the HPMC films were still elastic, resisting the shear deformation applied through the upper plate of the PPSR and causing wall slip resulting in artificially lower viscosity measurements to be recorded [40]. It is worth noting that the cast film of HPMC has slightly lower viscosity values compared to the compressed films probably due to the smoother surface of the cast film, further reducing the grip between the upper plate and the film during the application of the shear strain.

## 4. Conclusions

For the first time it was possible to demonstrate the feasibility of reconciling the complex viscosity data measured using DMA dual cantilever and PPSR. The extensive data of complex viscosity was reduced by representing each complex viscosity graph at each temperature step by power law parameters (***K*** and ***n***) such that each complex viscosity graph at one temperature is represented in one point in the ***K*** vs. tempetarure master curve and one point in the ***n*** vs. temperature counterpart. This allows the mechanical and rheological behaviour of the polymers to be successfully represented for the first time over a wide temperature range spanning across the Tg value. Moreover, this is the first study to demonstrate the possibility of using the complex viscosity equation below the T_g_ of amorphous polymers to gain an extra insight into their performance. In this study, the miscibility and overall effect of blending HPMC and HPC were successfully evaluated. The data generated suggests that these two polymers, though often employed together in pharmaceutical products, do not seem to be fully miscible. The mixtures indeed exhibit the individual thermal transition behaviour of both polymers in a pattern consistent with the fraction of each polymer in the mixture. The overall viscosity profile is however unique for each mixture.

The approaches described in this work could be used to optimise various polymeric solid dosage forms in terms of composition and process conditions—for example during hot melt extrusion or coating processes. While the use of complex viscosity might not be the usual function to represent the behaviour of the material in the glassy phase, it has been demonstrated here to be a reliable metric to span over the various phases. This approach offers an enhanced insight into the behaviour of the polymer over a wide range around the phase transition, making it easier to determine the ideal operating temperature and shear force range for a pharmaceutical process. 

## Figures and Tables

**Table 1:** Weight ratio of HPC and HPMC of polymer mixtures in the compressed films.

Composition Fraction (%*w*/*w*)	
HPC	HPMC
100	0
80	20
50	50
20	80
0	100

**Table 2:** Glass transition temperature of the HPC/HPMC compressed films as measured by the DMA. (±SD, n = 3).

HPC:HPMC Ratio (*w*/*w*)	Transition Temperature(s) (°C)
1:0	138.7 ± 0.5, 215.0 ± 1.1
8:2	140.3 ± 0.4, 223.5 ± 1.5
5:5	209.6 ± 1.2
2:8	217.6 ± 1.4
0:1	216.9 ± 3.2

**Table 3:** The ratio of the power law parameters for the compressed polymer mixtures calculated from the complex viscosity data measured by both DMA and PPSR.

HPC:HPMC (*w*:*w*)	Correlation Factor for *K* Value *K*_DMA_/*K*_PPSR_	Correlation Factor for *n* Value *n*_DMA_/*n*_PPSR_
1:0	12	1.5
8:2	12	1.5
5:5	12	1.5
2:8	4	1.5
0:1	9	1.5

## Data Availability

Experimental data produced during this work is held by the School of Pharmacy, University of Bradford. Requests for access should be made to a corresponding author.
